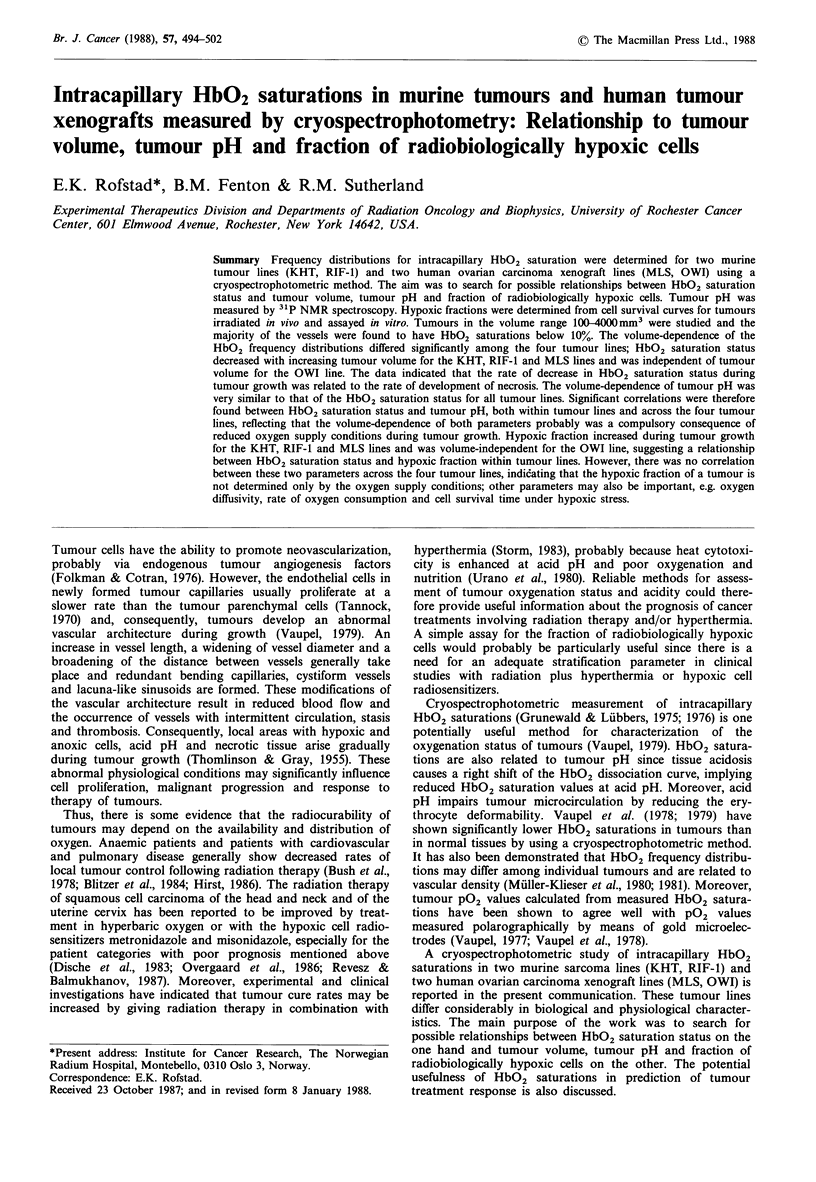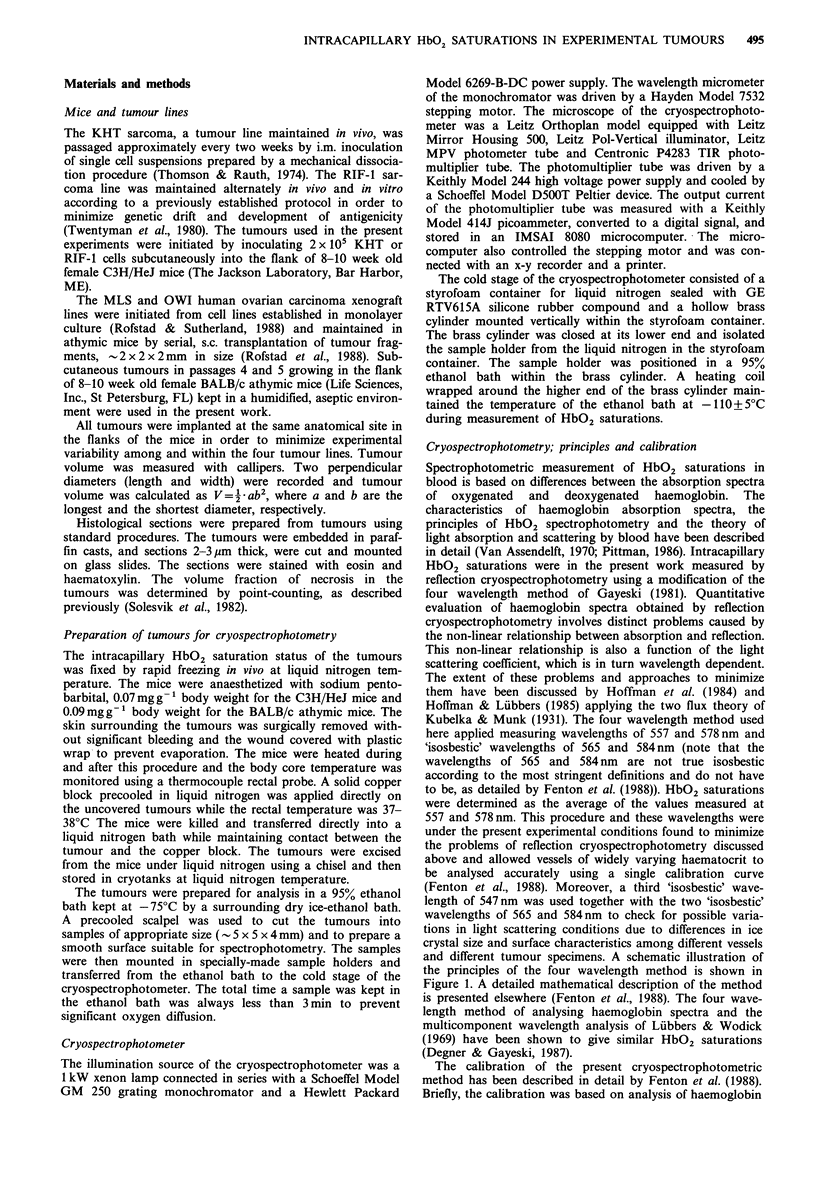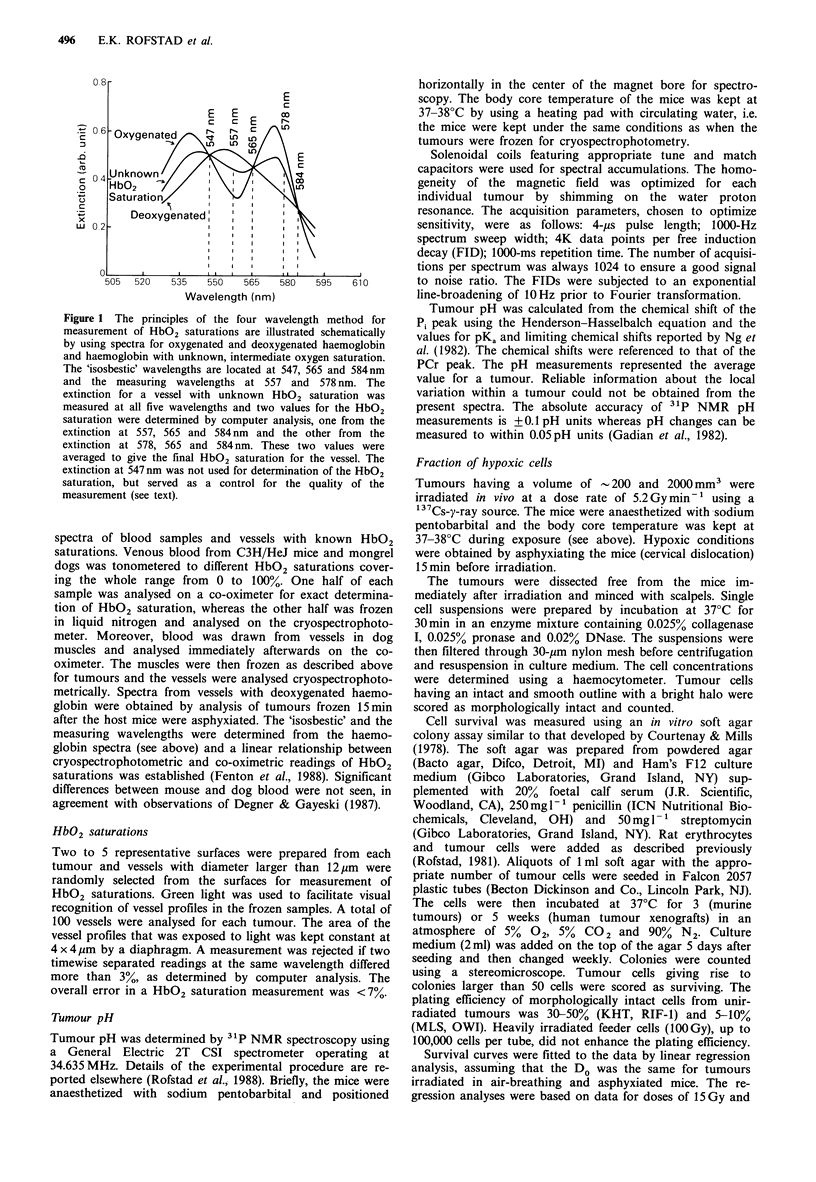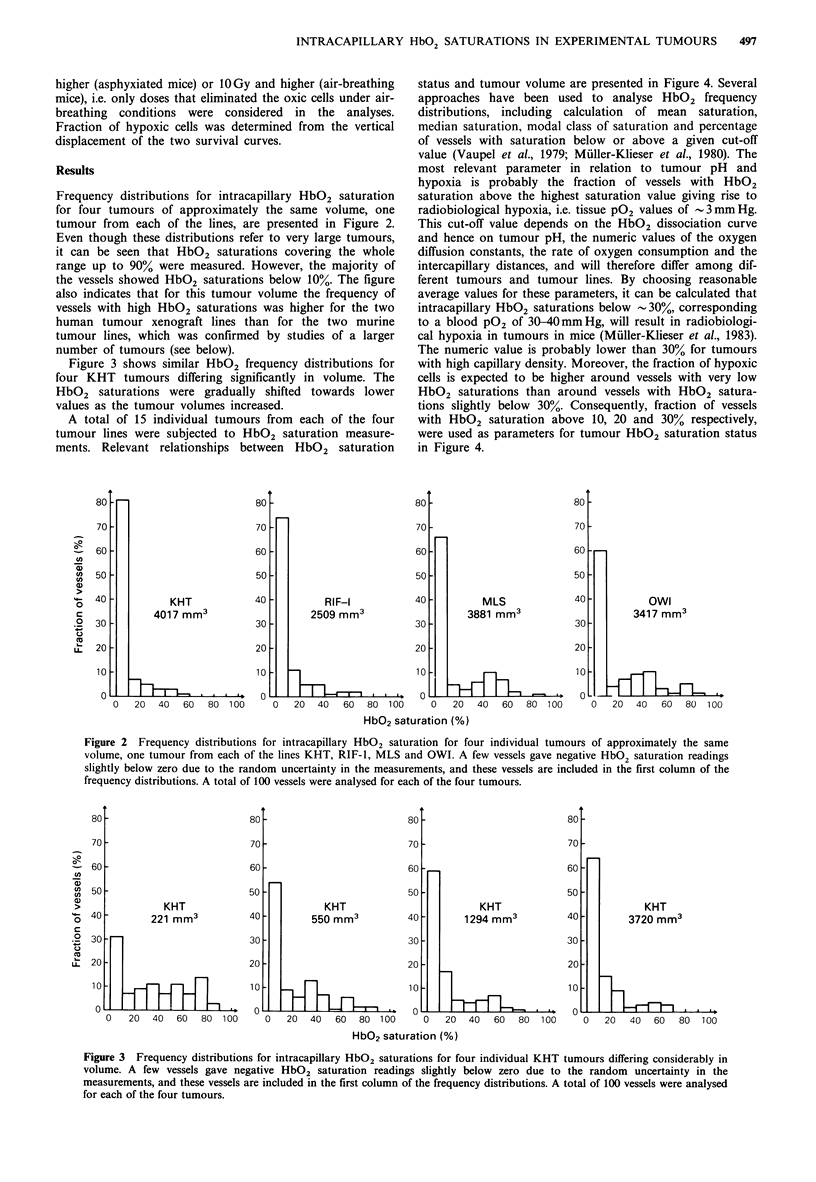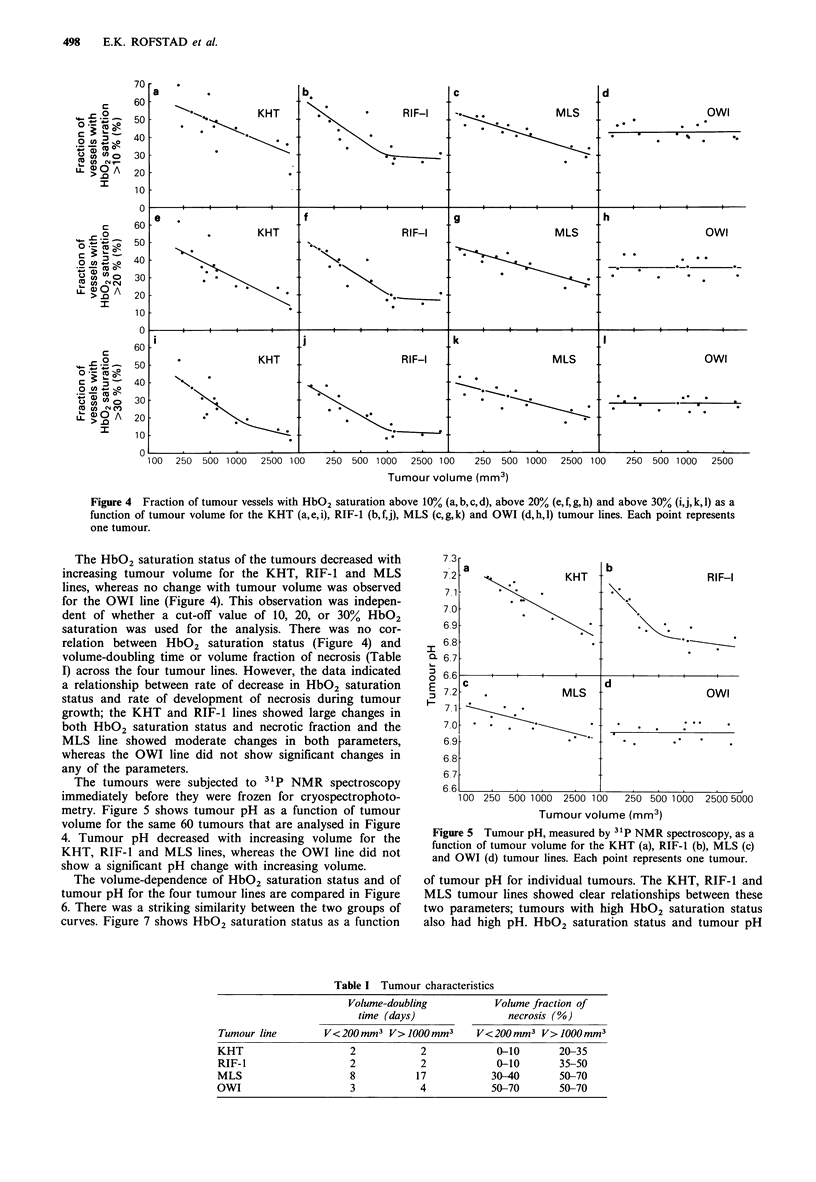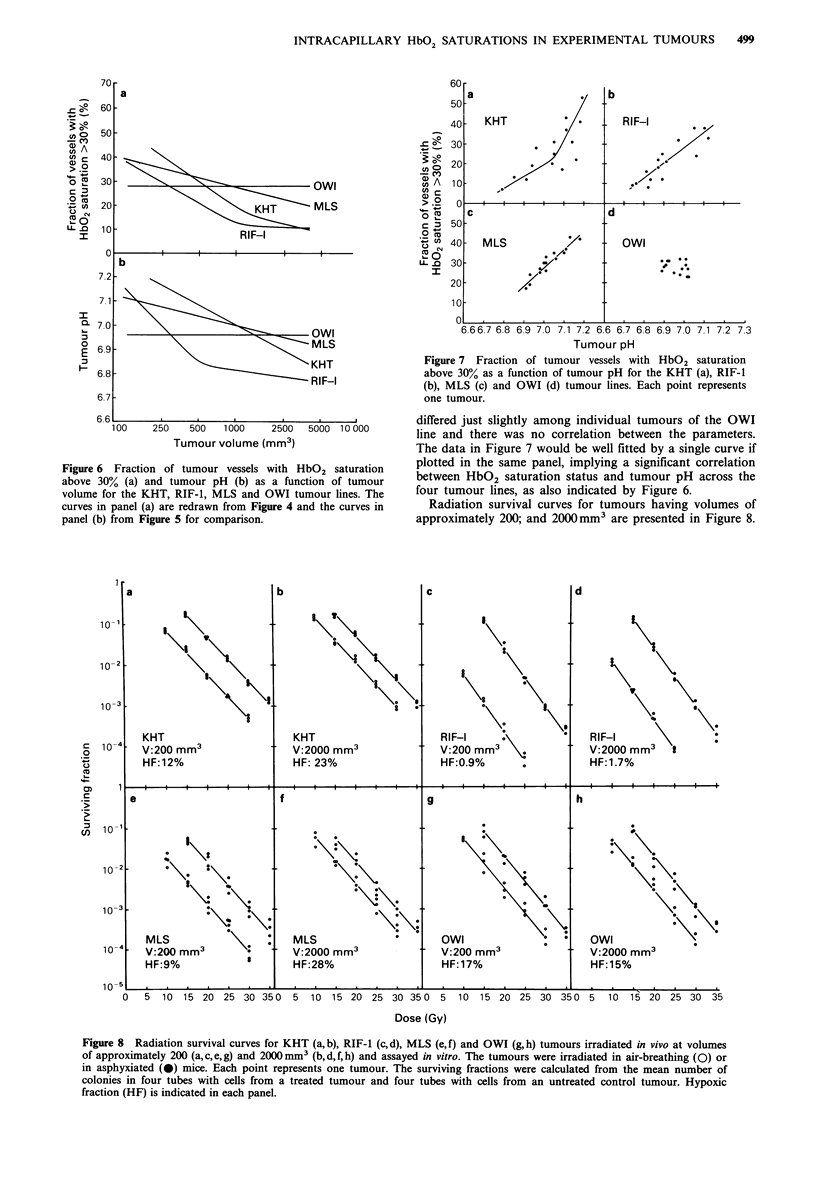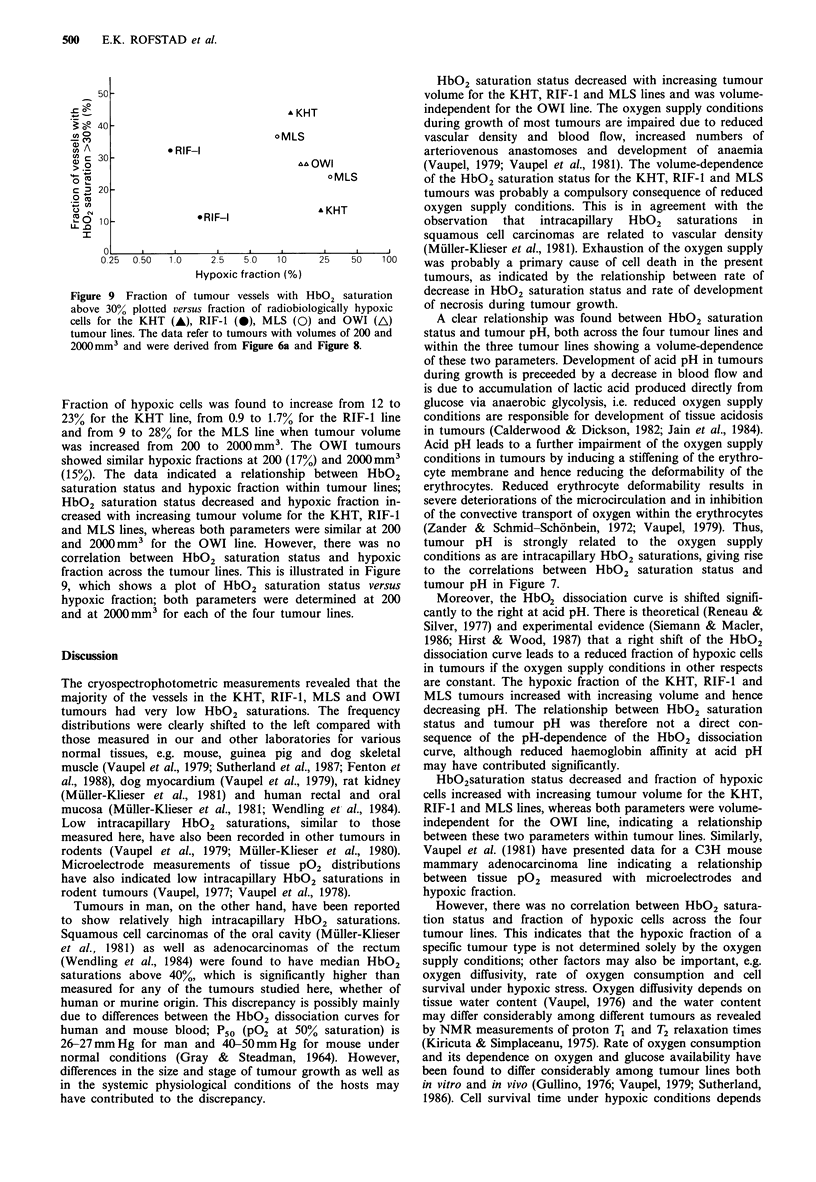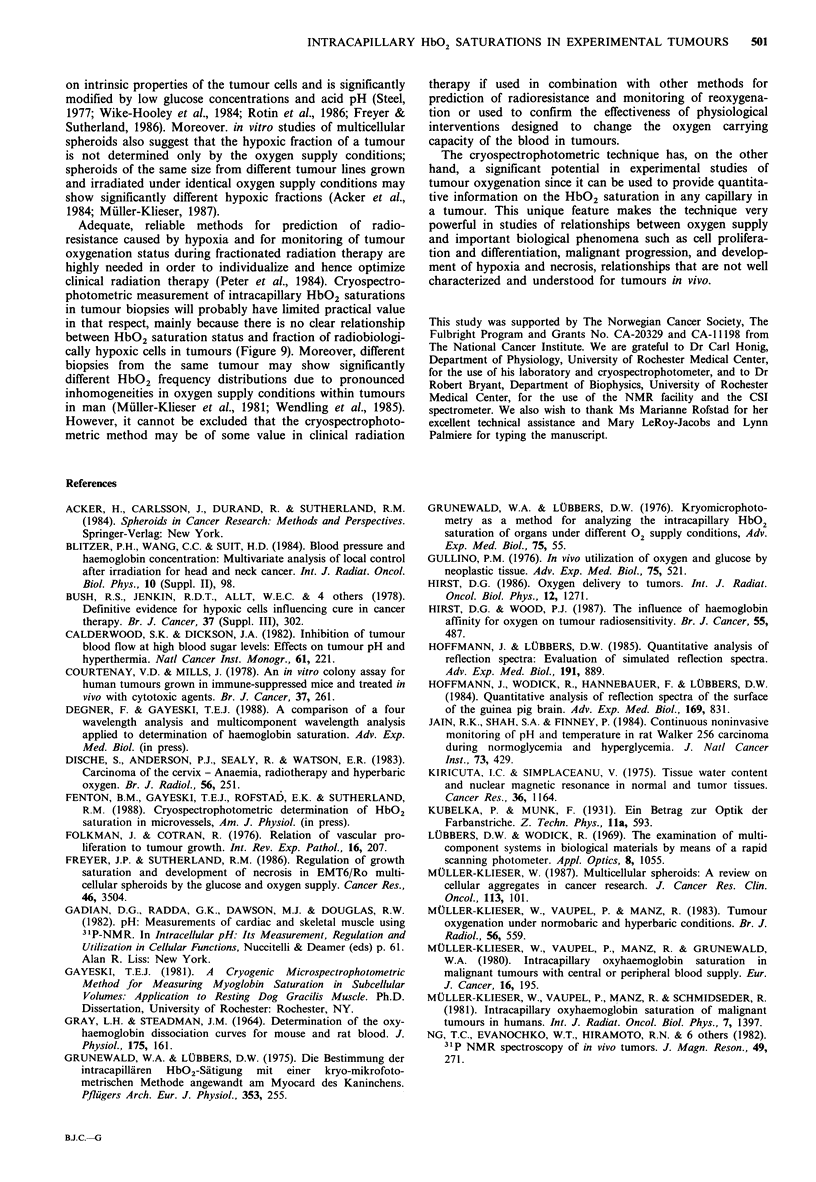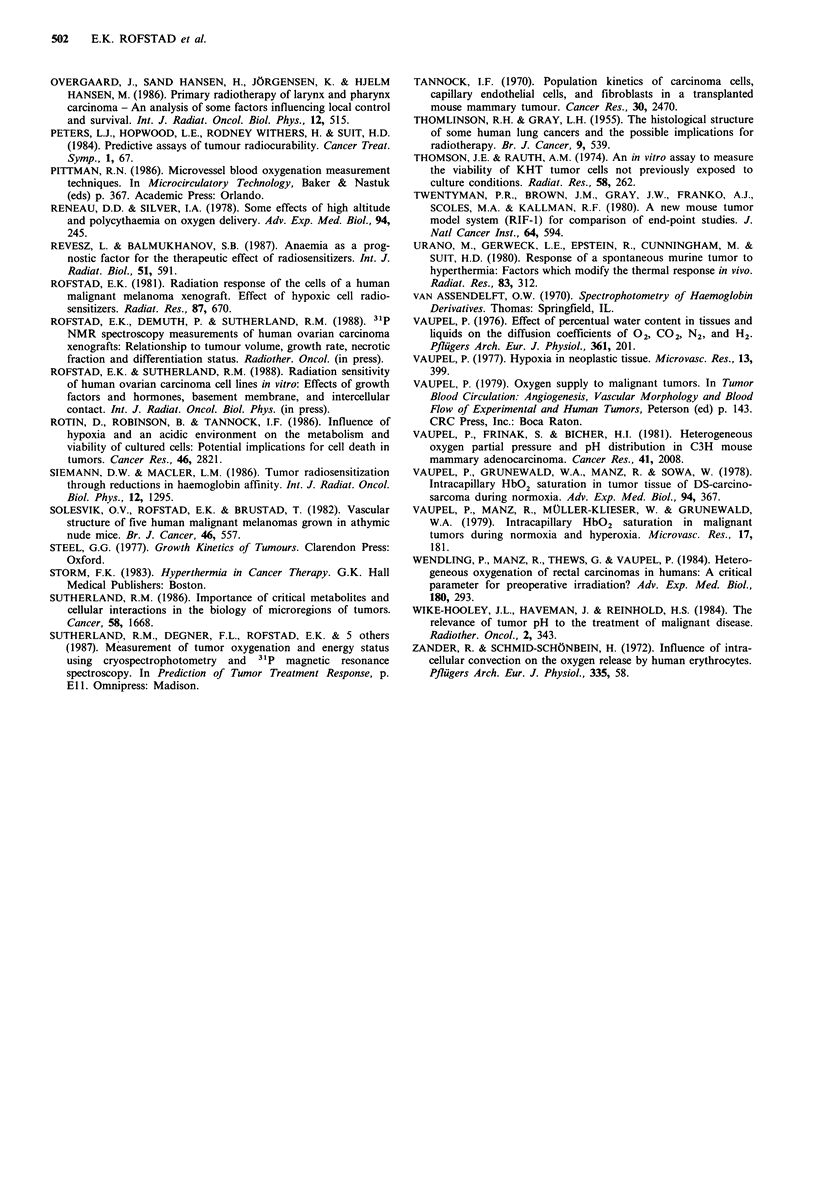# Intracapillary HbO2 saturations in murine tumours and human tumour xenografts measured by cryospectrophotometry: relationship to tumour volume, tumour pH and fraction of radiobiologically hypoxic cells.

**DOI:** 10.1038/bjc.1988.113

**Published:** 1988-05

**Authors:** E. K. Rofstad, B. M. Fenton, R. M. Sutherland

**Affiliations:** Experimental Therapeutics Division, University of Rochester Cancer Center, New York 14642.

## Abstract

Frequency distributions for intracapillary HbO2 saturation were determined for two murine tumour lines (KHT, RIF-1) and two human ovarian carcinoma xenograft lines (MLS, OWI) using a cryospectrophotometric method. The aim was to search for possible relationships between HbO2 saturation status and tumour volume, tumour pH and fraction of radiobiologically hypoxic cells. Tumour pH was measured by 31P NMR spectroscopy. Hypoxic fractions were determined from cell survival curves for tumours irradiated in vivo and assayed in vitro. Tumours in the volume range 100-4000 mm3 were studied and the majority of the vessels were found to have HbO2 saturations below 10%. The volume-dependence of the HbO2 frequency distributions differed significantly among the four tumour lines; HbO2 saturation status decreased with increasing tumour volume for the KHT, RIF-1 and MLS lines and was independent of tumour volume for the OWI line. The data indicated that the rate of decrease in HbO2 saturation status during tumour growth was related to the rate of development of necrosis. The volume-dependence of tumour pH was very similar to that of the HbO2 saturation status for all tumour lines. Significant correlations were therefore found between HbO2 saturation status and tumour pH, both within tumour lines and across the four tumour lines, reflecting that the volume-dependence of both parameters probably was a compulsory consequence of reduced oxygen supply conditions during tumour growth. Hypoxic fraction increased during tumour growth for the KHT, RIF-1 and MLS lines and was volume-independent for the OWI line, suggesting a relationship between HbO2 saturation status and hypoxic fraction within tumour lines. However, there was no correlation between these two parameters across the four tumour lines, indicating that the hypoxic fraction of a tumour is not determined only by the oxygen supply conditions; other parameters may also be important, e.g. oxygen diffusivity, rate of oxygen consumption and cell survival time under hypoxic stress.


Br. J. Cancer (1988), 57, 494–502 © The Macmillan Press Ltd., 1988

### Intracapillary $HbO_2$ saturations in murine tumours and human tumour xenografts measured by cryospectrophotometry: Relationship to tumour volume, tumour pH and fraction of radiobiologically hypoxic cells

E.K. Rofstad*, B.M. Fenton & R.M. Sutherland

*Experimental Therapeutics Division and Departments of Radiation Oncology and Biophysics, University of Rochester Cancer Center, 601 Elmwood Avenue, Rochester, New York 14642, USA.*

**Summary** Frequency distributions for intracapillary $HbO_2$ saturation were determined for two murine tumour lines (KHT, RIF-1) and two human ovarian carcinoma xenograft lines (MLS, OWI) using a cryospectrophotometric method. The aim was to search for possible relationships between $HbO_2$ saturation status and tumour volume, tumour pH and fraction of radiobiologically hypoxic cells. Tumour pH was measured by $^{31}P$ NMR spectroscopy. Hypoxic fractions were determined from cell survival curves for tumours irradiated *in vivo* and assayed *in vitro*. Tumours in the volume range 100–4000 $mm^3$ were studied and the majority of the vessels were found to have $HbO_2$ saturations below 10%. The volume-dependence of the $HbO_2$ frequency distributions differed significantly among the four tumour lines; $HbO_2$ saturation status decreased with increasing tumour volume for the KHT, RIF-1 and MLS lines and was independent of tumour volume for the OWI line. The data indicated that the rate of decrease in $HbO_2$ saturation status during tumour growth was related to the rate of development of necrosis. The volume-dependence of tumour pH was very similar to that of the $HbO_2$ saturation status for all tumour lines. Significant correlations were therefore found between $HbO_2$ saturation status and tumour pH, both within tumour lines and across the four tumour lines, reflecting that the volume-dependence of both parameters probably was a compulsory consequence of reduced oxygen supply conditions during tumour growth. Hypoxic fraction increased during tumour growth for the KHT, RIF-1 and MLS lines and was volume-independent for the OWI line, suggesting a relationship between $HbO_2$ saturation status and hypoxic fraction within tumour lines. However, there was no correlation between these two parameters across the four tumour lines, indicating that the hypoxic fraction of a tumour is not determined only by the oxygen supply conditions; other parameters may also be important, e.g. oxygen diffusivity, rate of oxygen consumption and cell survival time under hypoxic stress.

Tumour cells have the ability to promote neovascularization, probably via endogenous tumour angiogenesis factors (Folkman & Cotran, 1976). However, the endothelial cells in newly formed tumour capillaries usually proliferate at a slower rate than the tumour parenchymal cells (Tannock, 1970) and, consequently, tumours develop an abnormal vascular architecture during growth (Vaupel, 1979). An increase in vessel length, a widening of vessel diameter and a broadening of the distance between vessels generally take place and redundant bending capillaries, cystiform vessels and lacuna-like sinusoids are formed. These modifications of the vascular architecture result in reduced blood flow and the occurrence of vessels with intermittent circulation, stasis and thrombosis. Consequently, local areas with hypoxic and anoxic cells, acid pH and necrotic tissue arise gradually during tumour growth (Thomlinson & Gray, 1955). These abnormal physiological conditions may significantly influence cell proliferation, malignant progression and response to therapy of tumours.

Thus, there is some evidence that the radiocurability of tumours may depend on the availability and distribution of oxygen. Anaemic patients and patients with cardiovascular and pulmonary disease generally show decreased rates of local tumour control following radiation therapy (Bush *et al.*, 1978; Blitzer *et al.*, 1984; Hirst, 1986). The radiation therapy of squamous cell carcinoma of the head and neck and of the uterine cervix has been reported to be improved by treatment in hyperbaric oxygen or with the hypoxic cell radiosensitizers metronidazole and misonidazole, especially for the patient categories with poor prognosis mentioned above (Dische *et al.*, 1983; Overgaard *et al.*, 1986; Revesz & Balmukhanov, 1987). Moreover, experimental and clinical investigations have indicated that tumour cure rates may be increased by giving radiation therapy in combination with hyperthermia (Storm, 1983), probably because heat cytotoxicity is enhanced at acid pH and poor oxygenation and nutrition (Urano *et al.*, 1980). Reliable methods for assessment of tumour oxygenation status and acidity could therefore provide useful information about the prognosis of cancer treatments involving radiation therapy and/or hyperthermia. A simple assay for the fraction of radiobiologically hypoxic cells would probably be particularly useful since there is a need for an adequate stratification parameter in clinical studies with radiation plus hyperthermia or hypoxic cell radiosensitizers.

Cryospectrophotometric measurement of intracapillary $HbO_2$ saturations (Grunewald & Lübbers, 1975; 1976) is one potentially useful method for characterization of the oxygenation status of tumours (Vaupel, 1979). $HbO_2$ saturations are also related to tumour pH since tissue acidosis causes a right shift of the $HbO_2$ dissociation curve, implying reduced $HbO_2$ saturation values at acid pH. Moreover, acid pH impairs tumour microcirculation by reducing the erythrocyte deformability. Vaupel *et al.* (1978; 1979) have shown significantly lower $HbO_2$ saturations in tumours than in normal tissues by using a cryospectrophotometric method. It has also been demonstrated that $HbO_2$ frequency distributions may differ among individual tumours and are related to vascular density (Müller-Klieser *et al.*, 1980; 1981). Moreover, tumour $pO_2$ values calculated from measured $HbO_2$ saturations have been shown to agree well with $pO_2$ values measured polarographically by means of gold microelectrodes (Vaupel, 1977; Vaupel *et al.*, 1978).

A cryospectrophotometric study of intracapillary $HbO_2$ saturations in two murine sarcoma lines (KHT, RIF-1) and two human ovarian carcinoma xenograft lines (MLS, OWI) is reported in the present communication. These tumour lines differ considerably in biological and physiological characteristics. The main purpose of the work was to search for possible relationships between $HbO_2$ saturation status on the one hand and tumour volume, tumour pH and fraction of radiobiologically hypoxic cells on the other. The potential usefulness of $HbO_2$ saturations in prediction of tumour treatment response is also discussed.

*Present address: Institute for Cancer Research, The Norwegian Radium Hospital, Montebello, 0310 Oslo 3, Norway.
Correspondence: E.K. Rofstad.
Received 23 October 1987; and in revised form 8 January 1988.

### Materials and methods

### *Mice and tumour lines*

The KHT sarcoma, a tumour line maintained *in vivo*, was passaged approximately every two weeks by i.m. inoculation of single cell suspensions prepared by a mechanical dissociation procedure (Thomson & Rauth, 1974). The RIF-1 sarcoma line was maintained alternately *in vivo* and *in vitro* according to a previously established protocol in order to minimize genetic drift and development of antigenicity (Twentyman *et al.*, 1980). The tumours used in the present experiments were initiated by inoculating $2 \times 10^5$ KHT or RIF-1 cells subcutaneously into the flank of 8–10 week old female C3H/HeJ mice (The Jackson Laboratory, Bar Harbor, ME).

The MLS and OWI human ovarian carcinoma xenograft lines were initiated from cell lines established in monolayer culture (Rofstad & Sutherland, 1988) and maintained in athymic mice by serial, s.c. transplantation of tumour fragments, $\sim 2 \times 2 \times 2$ mm in size (Rofstad *et al.*, 1988). Subcutaneous tumours in passages 4 and 5 growing in the flank of 8–10 week old female BALB/c athymic mice (Life Sciences, Inc., St Petersburg, FL) kept in a humidified, aseptic environment were used in the present work.

All tumours were implanted at the same anatomical site in the flanks of the mice in order to minimize experimental variability among and within the four tumour lines. Tumour volume was measured with callipers. Two perpendicular diameters (length and width) were recorded and tumour volume was calculated as $V = \frac{1}{2} \cdot ab^2$, where $a$ and $b$ are the longest and the shortest diameter, respectively.

Histological sections were prepared from tumours using standard procedures. The tumours were embedded in paraffin casts, and sections 2–3 $\mu$m thick, were cut and mounted on glass slides. The sections were stained with eosin and haematoxylin. The volume fraction of necrosis in the tumours was determined by point-counting, as described previously (Solesvik *et al.*, 1982).

### *Preparation of tumours for cryospectrophotometry*

The intracapillary $HbO_2$ saturation status of the tumours was fixed by rapid freezing *in vivo* at liquid nitrogen temperature. The mice were anaesthetized with sodium pentobarbital, 0.07 mg $g^{-1}$ body weight for the C3H/HeJ mice and 0.09 mg $g^{-1}$ body weight for the BALB/c athymic mice. The skin surrounding the tumours was surgically removed without significant bleeding and the wound covered with plastic wrap to prevent evaporation. The mice were heated during and after this procedure and the body core temperature was monitored using a thermocouple rectal probe. A solid copper block precooled in liquid nitrogen was applied directly on the uncovered tumours while the rectal temperature was 37–38°C The mice were killed and transferred directly into a liquid nitrogen bath while maintaining contact between the tumour and the copper block. The tumours were excised from the mice under liquid nitrogen using a chisel and then stored in cryotanks at liquid nitrogen temperature.

The tumours were prepared for analysis in a 95% ethanol bath kept at −75°C by a surrounding dry ice-ethanol bath. A precooled scalpel was used to cut the tumours into samples of appropriate size ($\sim 5 \times 5 \times 4$ mm) and to prepare a smooth surface suitable for spectrophotometry. The samples were then mounted in specially-made sample holders and transferred from the ethanol bath to the cold stage of the cryospectrophotometer. The total time a sample was kept in the ethanol bath was always less than 3 min to prevent significant oxygen diffusion.

### *Cryospectrophotometer*

The illumination source of the cryospectrophotometer was a 1 kW xenon lamp connected in series with a Schoeffel Model GM 250 grating monochromator and a Hewlett Packard Model 6269-B-DC power supply. The wavelength micrometer of the monochromator was driven by a Hayden Model 7532 stepping motor. The microscope of the cryospectrophotometer was a Leitz Orthoplan model equipped with Leitz Mirror Housing 500, Leitz Pol-Vertical illuminator, Leitz MPV photometer tube and Centronic P4283 TIR photomultiplier tube. The photomultiplier tube was driven by a Keithly Model 244 high voltage power supply and cooled by a Schoeffel Model D500T Peltier device. The output current of the photomultiplier tube was measured with a Keithly Model 414J picoammeter, converted to a digital signal, and stored in an IMSAI 8080 microcomputer. The microcomputer also controlled the stepping motor and was connected with an x-y recorder and a printer.

The cold stage of the cryospectrophotometer consisted of a styrofoam container for liquid nitrogen sealed with GE RTV615A silicone rubber compound and a hollow brass cylinder mounted vertically within the styrofoam container. The brass cylinder was closed at its lower end and isolated the sample holder from the liquid nitrogen in the styrofoam container. The sample holder was positioned in a 95% ethanol bath within the brass cylinder. A heating coil wrapped around the higher end of the brass cylinder maintained the temperature of the ethanol bath at $-110 \pm 5$°C during measurement of $HbO_2$ saturations.

### *Cryospectrophotometry; principles and calibration*

Spectrophotometric measurement of $HbO_2$ saturations in blood is based on differences between the absorption spectra of oxygenated and deoxygenated haemoglobin. The characteristics of haemoglobin absorption spectra, the principles of $HbO_2$ spectrophotometry and the theory of light absorption and scattering by blood have been described in detail (Van Assendelft, 1970; Pittman, 1986). Intracapillary $HbO_2$ saturations were in the present work measured by reflection cryospectrophotometry using a modification of the four wavelength method of Gayeski (1981). Quantitative evaluation of haemoglobin spectra obtained by reflection cryospectrophotometry involves distinct problems caused by the non-linear relationship between absorption and reflection. This non-linear relationship is also a function of the light scattering coefficient, which is in turn wavelength dependent. The extent of these problems and approaches to minimize them have been discussed by Hoffman *et al.* (1984) and Hoffman & Lübbers (1985) applying the two flux theory of Kubelka & Munk (1931). The four wavelength method used here applied measuring wavelengths of 557 and 578 nm and 'isosbestic' wavelengths of 565 and 584 nm (note that the wavelengths of 565 and 584 nm are not true isosbestic according to the most stringent definitions and do not have to be, as detailed by Fenton *et al.* (1988)). $HbO_2$ saturations were determined as the average of the values measured at 557 and 578 nm. This procedure and these wavelengths were under the present experimental conditions found to minimize the problems of reflection cryospectrophotometry discussed above and allowed vessels of widely varying haematocrit to be analysed accurately using a single calibration curve (Fenton *et al.*, 1988). Moreover, a third 'isosbestic' wavelength of 547 nm was used together with the two 'isosbestic' wavelengths of 565 and 584 nm to check for possible variations in light scattering conditions due to differences in ice crystal size and surface characteristics among different vessels and different tumour specimens. A schematic illustration of the principles of the four wavelength method is shown in Figure 1. A detailed mathematical description of the method is presented elsewhere (Fenton *et al.*, 1988). The four wavelength method of analysing haemoglobin spectra and the multicomponent wavelength analysis of Lübbers & Wodick (1969) have been shown to give similar $HbO_2$ saturations (Degner & Gayeski, 1987).

The calibration of the present cryospectrophotometric method has been described in detail by Fenton *et al.* (1988). Briefly, the calibration was based on analysis of haemoglobin

**Figure 1** The principles of the four wavelength method for measurement of $HbO_2$ saturations are illustrated schematically by using spectra for oxygenated and deoxygenated haemoglobin and haemoglobin with unknown, intermediate oxygen saturation. The 'isosbestic' wavelengths are located at 547, 565 and 584 nm and the measuring wavelengths at 557 and 578 nm. The extinction for a vessel with unknown $HbO_2$ saturation was measured at all five wavelengths and two values for the $HbO_2$ saturation were determined by computer analysis, one from the extinction at 557, 565 and 584 nm and the other from the extinction at 578, 565 and 584 nm. These two values were averaged to give the final $HbO_2$ saturation for the vessel. The extinction at 547 nm was not used for determination of the $HbO_2$ saturation, but served as a control for the quality of the measurement (see text).

spectra of blood samples and vessels with known $HbO_2$ saturations. Venous blood from C3H/HeJ mice and mongrel dogs was tonometered to different $HbO_2$ saturations covering the whole range from 0 to 100%. One half of each sample was analysed on a co-oximeter for exact determination of $HbO_2$ saturation, whereas the other half was frozen in liquid nitrogen and analysed on the cryospectrophotometer. Moreover, blood was drawn from vessels in dog muscles and analysed immediately afterwards on the co-oximeter. The muscles were then frozen as described above for tumours and the vessels were analysed cryospectrophotometrically. Spectra from vessels with deoxygenated haemoglobin were obtained by analysis of tumours frozen 15 min after the host mice were asphyxiated. The 'isosbestic' and the measuring wavelengths were determined from the haemoglobin spectra (see above) and a linear relationship between cryospectrophotometric and co-oximetric readings of $HbO_2$ saturations was established (Fenton *et al.*, 1988). Significant differences between mouse and dog blood were not seen, in agreement with observations of Degner & Gayeski (1987).

*$HbO_2$ saturations*

Two to 5 representative surfaces were prepared from each tumour and vessels with diameter larger than 12 $\mu$m were randomly selected from the surfaces for measurement of $HbO_2$ saturations. Green light was used to facilitate visual recognition of vessel profiles in the frozen samples. A total of 100 vessels were analysed for each tumour. The area of the vessel profiles that was exposed to light was kept constant at $4 \times 4\,\mu$m by a diaphragm. A measurement was rejected if two timewise separated readings at the same wavelength differed more than 3%, as determined by computer analysis. The overall error in a $HbO_2$ saturation measurement was <7%.

*Tumour pH*

Tumour pH was determined by $^{31}$P NMR spectroscopy using a General Electric 2T CSI spectrometer operating at 34.635 MHz. Details of the experimental procedure are reported elsewhere (Rofstad *et al.*, 1988). Briefly, the mice were anaesthetized with sodium pentobarbital and positioned horizontally in the center of the magnet bore for spectroscopy. The body core temperature of the mice was kept at 37–38°C by using a heating pad with circulating water, i.e. the mice were kept under the same conditions as when the tumours were frozen for cryospectrophotometry.

Solenoidal coils featuring appropriate tune and match capacitors were used for spectral accumulations. The homogeneity of the magnetic field was optimized for each individual tumour by shimming on the water proton resonance. The acquisition parameters, chosen to optimize sensitivity, were as follows: 4-$\mu$s pulse length; 1000-Hz spectrum sweep width; 4K data points per free induction decay (FID); 1000-ms repetition time. The number of acquisitions per spectrum was always 1024 to ensure a good signal to noise ratio. The FIDs were subjected to an exponential line-broadening of 10 Hz prior to Fourier transformation.

Tumour pH was calculated from the chemical shift of the $P_i$ peak using the Henderson–Hasselbalch equation and the values for $pK_a$ and limiting chemical shifts reported by Ng *et al.* (1982). The chemical shifts were referenced to that of the PCr peak. The pH measurements represented the average value for a tumour. Reliable information about the local variation within a tumour could not be obtained from the present spectra. The absolute accuracy of $^{31}$P NMR pH measurements is $\pm 0.1$ pH units whereas pH changes can be measured to within 0.05 pH units (Gadian *et al.*, 1982).

*Fraction of hypoxic cells*

Tumours having a volume of ~200 and 2000 mm$^3$ were irradiated *in vivo* at a dose rate of 5.2 Gy min$^{-1}$ using a $^{137}$Cs-$\gamma$-ray source. The mice were anaesthetized with sodium pentobarbital and the body core temperature was kept at 37–38°C during exposure (see above). Hypoxic conditions were obtained by asphyxiating the mice (cervical dislocation) 15 min before irradiation.

The tumours were dissected free from the mice immediately after irradiation and minced with scalpels. Single cell suspensions were prepared by incubation at 37°C for 30 min in an enzyme mixture containing 0.025% collagenase I, 0.025% pronase and 0.02% DNase. The suspensions were then filtered through 30-$\mu$m nylon mesh before centrifugation and resuspension in culture medium. The cell concentrations were determined using a haemocytometer. Tumour cells having an intact and smooth outline with a bright halo were scored as morphologically intact and counted.

Cell survival was measured using an *in vitro* soft agar colony assay similar to that developed by Courtenay & Mills (1978). The soft agar was prepared from powdered agar (Bacto agar, Difco, Detroit, MI) and Ham's F12 culture medium (Gibco Laboratories, Grand Island, NY) supplemented with 20% foetal calf serum (J.R. Scientific, Woodland, CA), 250 mg l$^{-1}$ penicillin (ICN Nutritional Biochemicals, Cleveland, OH) and 50 mg l$^{-1}$ streptomycin (Gibco Laboratories, Grand Island, NY). Rat erythrocytes and tumour cells were added as described previously (Rofstad, 1981). Aliquots of 1 ml soft agar with the appropriate number of tumour cells were seeded in Falcon 2057 plastic tubes (Becton Dickinson and Co., Lincoln Park, NJ). The cells were then incubated at 37°C for 3 (murine tumours) or 5 weeks (human tumour xenografts) in an atmosphere of 5% $O_2$, 5% $CO_2$ and 90% $N_2$. Culture medium (2 ml) was added on the top of the agar 5 days after seeding and then changed weekly. Colonies were counted using a stereomicroscope. Tumour cells giving rise to colonies larger than 50 cells were scored as surviving. The plating efficiency of morphologically intact cells from unirradiated tumours was 30–50% (KHT, RIF-1) and 5–10% (MLS, OWI). Heavily irradiated feeder cells (100 Gy), up to 100,000 cells per tube, did not enhance the plating efficiency.

Survival curves were fitted to the data by linear regression analysis, assuming that the $D_0$ was the same for tumours irradiated in air-breathing and asphyxiated mice. The regression analyses were based on data for doses of 15 Gy and higher (asphyxiated mice) or 10 Gy and higher (air-breathing mice), i.e. only doses that eliminated the oxic cells under air-breathing conditions were considered in the analyses. Fraction of hypoxic cells was determined from the vertical displacement of the two survival curves.

### Results

Frequency distributions for intracapillary $HbO_2$ saturation for four tumours of approximately the same volume, one tumour from each of the lines, are presented in Figure 2. Even though these distributions refer to very large tumours, it can be seen that $HbO_2$ saturations covering the whole range up to 90% were measured. However, the majority of the vessels showed $HbO_2$ saturations below 10%. The figure also indicates that for this tumour volume the frequency of vessels with high $HbO_2$ saturations was higher for the two human tumour xenograft lines than for the two murine tumour lines, which was confirmed by studies of a larger number of tumours (see below).

Figure 3 shows similar $HbO_2$ frequency distributions for four KHT tumours differing significantly in volume. The $HbO_2$ saturations were gradually shifted towards lower values as the tumour volumes increased.

A total of 15 individual tumours from each of the four tumour lines were subjected to $HbO_2$ saturation measurements. Relevant relationships between $HbO_2$ saturation status and tumour volume are presented in Figure 4. Several approaches have been used to analyse $HbO_2$ frequency distributions, including calculation of mean saturation, median saturation, modal class of saturation and percentage of vessels with saturation below or above a given cut-off value (Vaupel *et al.*, 1979; Müller-Klieser *et al.*, 1980). The most relevant parameter in relation to tumour pH and hypoxia is probably the fraction of vessels with $HbO_2$ saturation above the highest saturation value giving rise to radiobiological hypoxia, i.e. tissue $pO_2$ values of ~3 mm Hg. This cut-off value depends on the $HbO_2$ dissociation curve and hence on tumour pH, the numeric values of the oxygen diffusion constants, the rate of oxygen consumption and the intercapillary distances, and will therefore differ among different tumours and tumour lines. By choosing reasonable average values for these parameters, it can be calculated that intracapillary $HbO_2$ saturations below ~30%, corresponding to a blood $pO_2$ of 30–40 mm Hg, will result in radiobiological hypoxia in tumours in mice (Müller-Klieser *et al.*, 1983). The numeric value is probably lower than 30% for tumours with high capillary density. Moreover, the fraction of hypoxic cells is expected to be higher around vessels with very low $HbO_2$ saturations than around vessels with $HbO_2$ saturations slightly below 30%. Consequently, fraction of vessels with $HbO_2$ saturation above 10, 20 and 30% respectively, were used as parameters for tumour $HbO_2$ saturation status in Figure 4.

**Figure 2** Frequency distributions for intracapillary $HbO_2$ saturation for four individual tumours of approximately the same volume, one tumour from each of the lines KHT, RIF-1, MLS and OWI. A few vessels gave negative $HbO_2$ saturation readings slightly below zero due to the random uncertainty in the measurements, and these vessels are included in the first column of the frequency distributions. A total of 100 vessels were analysed for each of the four tumours.

**Figure 3** Frequency distributions for intracapillary $HbO_2$ saturations for four individual KHT tumours differing considerably in volume. A few vessels gave negative $HbO_2$ saturation readings slightly below zero due to the random uncertainty in the measurements, and these vessels are included in the first column of the frequency distributions. A total of 100 vessels were analysed for each of the four tumours.

**Figure 4** Fraction of tumour vessels with $HbO_2$ saturation above 10% (a,b,c,d), above 20% (e,f,g,h) and above 30% (i,j,k,l) as a function of tumour volume for the KHT (a,e,i), RIF-1 (b,f,j), MLS (c,g,k) and OWI (d,h,l) tumour lines. Each point represents one tumour.

The $HbO_2$ saturation status of the tumours decreased with increasing tumour volume for the KHT, RIF-1 and MLS lines, whereas no change with tumour volume was observed for the OWI line (Figure 4). This observation was independent of whether a cut-off value of 10, 20, or 30% $HbO_2$ saturation was used for the analysis. There was no correlation between $HbO_2$ saturation status (Figure 4) and volume-doubling time or volume fraction of necrosis (Table I) across the four tumour lines. However, the data indicated a relationship between rate of decrease in $HbO_2$ saturation status and rate of development of necrosis during tumour growth; the KHT and RIF-1 lines showed large changes in both $HbO_2$ saturation status and necrotic fraction and the MLS line showed moderate changes in both parameters, whereas the OWI line did not show significant changes in any of the parameters.

The tumours were subjected to $^{31}P$ NMR spectroscopy immediately before they were frozen for cryospectrophotometry. Figure 5 shows tumour pH as a function of tumour volume for the same 60 tumours that are analysed in Figure 4. Tumour pH decreased with increasing volume for the KHT, RIF-1 and MLS lines, whereas the OWI line did not show a significant pH change with increasing volume.

The volume-dependence of $HbO_2$ saturation status and of tumour pH for the four tumour lines are compared in Figure 6. There was a striking similarity between the two groups of curves. Figure 7 shows $HbO_2$ saturation status as a function

**Figure 5** Tumour pH, measured by $^{31}P$ NMR spectroscopy, as a function of tumour volume for the KHT (a), RIF-1 (b), MLS (c) and OWI (d) tumour lines. Each point represents one tumour.

of tumour pH for individual tumours. The KHT, RIF-1 and MLS tumour lines showed clear relationships between these two parameters; tumours with high $HbO_2$ saturation status also had high pH. $HbO_2$ saturation status and tumour pH

**Table I** Tumour characteristics

| | *Volume-doubling time (days)* | | *Volume fraction of necrosis (%)* | |
|---|---|---|---|---|
| *Tumour line* | *V<200 mm³* | *V>1000 mm³* | *V<200 mm³* | *V>1000 mm³* |
| KHT | 2 | 2 | 0–10 | 20–35 |
| RIF-1 | 2 | 2 | 0–10 | 35–50 |
| MLS | 8 | 17 | 30–40 | 50–70 |
| OWI | 3 | 4 | 50–70 | 50–70 |

**Figure 6** Fraction of tumour vessels with $HbO_2$ saturation above 30% (a) and tumour pH (b) as a function of tumour volume for the KHT, RIF-1, MLS and OWI tumour lines. The curves in panel (a) are redrawn from **Figure 4** and the curves in panel (b) from **Figure 5** for comparison.

**Figure 7** Fraction of tumour vessels with $HbO_2$ saturation above 30% as a function of tumour pH for the KHT (a), RIF-1 (b), MLS (c) and OWI (d) tumour lines. Each point represents one tumour.

differed just slightly among individual tumours of the OWI line and there was no correlation between the parameters. The data in Figure 7 would be well fitted by a single curve if plotted in the same panel, implying a significant correlation between $HbO_2$ saturation status and tumour pH across the four tumour lines, as also indicated by Figure 6.

Radiation survival curves for tumours having volumes of approximately 200; and 2000 mm$^3$ are presented in Figure 8.

**Figure 8** Radiation survival curves for KHT (a, b), RIF-1 (c, d), MLS (e, f) and OWI (g, h) tumours irradiated *in vivo* at volumes of approximately 200 (a, c, e, g) and 2000 mm$^3$ (b, d, f, h) and assayed *in vitro*. The tumours were irradiated in air-breathing (○) or in asphyxiated (●) mice. Each point represents one tumour. The surviving fractions were calculated from the mean number of colonies in four tubes with cells from a treated tumour and four tubes with cells from an untreated control tumour. Hypoxic fraction (HF) is indicated in each panel.

**Figure 9** Fraction of tumour vessels with $HbO_2$ saturation above 30% plotted *versus* fraction of radiobiologically hypoxic cells for the KHT (▲), RIF-1 (●), MLS (○) and OWI (△) tumour lines. The data refer to tumours with volumes of 200 and 2000 $mm^3$ and were derived from **Figure 6a** and **Figure 8**.

Fraction of hypoxic cells was found to increase from 12 to 23% for the KHT line, from 0.9 to 1.7% for the RIF-1 line and from 9 to 28% for the MLS line when tumour volume was increased from 200 to 2000 $mm^3$. The OWI tumours showed similar hypoxic fractions at 200 (17%) and 2000 $mm^3$ (15%). The data indicated a relationship between $HbO_2$ saturation status and hypoxic fraction within tumour lines; $HbO_2$ saturation status decreased and hypoxic fraction increased with increasing tumour volume for the KHT, RIF-1 and MLS lines, whereas both parameters were similar at 200 and 2000 $mm^3$ for the OWI line. However, there was no correlation between $HbO_2$ saturation status and hypoxic fraction across the tumour lines. This is illustrated in Figure 9, which shows a plot of $HbO_2$ saturation status *versus* hypoxic fraction; both parameters were determined at 200 and at 2000 $mm^3$ for each of the four tumour lines.

### Discussion

The cryospectrophotometric measurements revealed that the majority of the vessels in the KHT, RIF-1, MLS and OWI tumours had very low $HbO_2$ saturations. The frequency distributions were clearly shifted to the left compared with those measured in our and other laboratories for various normal tissues, e.g. mouse, guinea pig and dog skeletal muscle (Vaupel *et al.*, 1979; Sutherland *et al.*, 1987; Fenton *et al.*, 1988), dog myocardium (Vaupel *et al.*, 1979), rat kidney (Müller-Klieser *et al.*, 1981) and human rectal and oral mucosa (Müller-Klieser *et al.*, 1981; Wendling *et al.*, 1984). Low intracapillary $HbO_2$ saturations, similar to those measured here, have also been recorded in other tumours in rodents (Vaupel *et al.*, 1979; Müller-Klieser *et al.*, 1980). Microelectrode measurements of tissue $pO_2$ distributions have also indicated low intracapillary $HbO_2$ saturations in rodent tumours (Vaupel, 1977; Vaupel *et al.*, 1978).

Tumours in man, on the other hand, have been reported to show relatively high intracapillary $HbO_2$ saturations. Squamous cell carcinomas of the oral cavity (Müller-Klieser *et al.*, 1981) as well as adenocarcinomas of the rectum (Wendling *et al.*, 1984) were found to have median $HbO_2$ saturations above 40%, which is significantly higher than measured for any of the tumours studied here, whether of human or murine origin. This discrepancy is possibly mainly due to differences between the $HbO_2$ dissociation curves for human and mouse blood; $P_{50}$ ($pO_2$ at 50% saturation) is 26–27 mm Hg for man and 40–50 mm Hg for mouse under normal conditions (Gray & Steadman, 1964). However, differences in the size and stage of tumour growth as well as in the systemic physiological conditions of the hosts may have contributed to the discrepancy.

$HbO_2$ saturation status decreased with increasing tumour volume for the KHT, RIF-1 and MLS lines and was volume-independent for the OWI line. The oxygen supply conditions during growth of most tumours are impaired due to reduced vascular density and blood flow, increased numbers of arteriovenous anastomoses and development of anaemia (Vaupel, 1979; Vaupel *et al.*, 1981). The volume-dependence of the $HbO_2$ saturation status for the KHT, RIF-1 and MLS tumours was probably a compulsory consequence of reduced oxygen supply conditions. This is in agreement with the observation that intracapillary $HbO_2$ saturations in squamous cell carcinomas are related to vascular density (Müller-Klieser *et al.*, 1981). Exhaustion of the oxygen supply was probably a primary cause of cell death in the present tumours, as indicated by the relationship between rate of decrease in $HbO_2$ saturation status and rate of development of necrosis during tumour growth.

A clear relationship was found between $HbO_2$ saturation status and tumour pH, both across the four tumour lines and within the three tumour lines showing a volume-dependence of these two parameters. Development of acid pH in tumours during growth is preceeded by a decrease in blood flow and is due to accumulation of lactic acid produced directly from glucose via anaerobic glycolysis, i.e. reduced oxygen supply conditions are responsible for development of tissue acidosis in tumours (Calderwood & Dickson, 1982; Jain *et al.*, 1984). Acid pH leads to a further impairment of the oxygen supply conditions in tumours by inducing a stiffening of the erythrocyte membrane and hence reducing the deformability of the erythrocytes. Reduced erythrocyte deformability results in severe deteriorations of the microcirculation and in inhibition of the convective transport of oxygen within the erythrocytes (Zander & Schmid-Schönbein, 1972; Vaupel, 1979). Thus, tumour pH is strongly related to the oxygen supply conditions as are intracapillary $HbO_2$ saturations, giving rise to the correlations between $HbO_2$ saturation status and tumour pH in Figure 7.

Moreover, the $HbO_2$ dissociation curve is shifted significantly to the right at acid pH. There is theoretical (Reneau & Silver, 1977) and experimental evidence (Siemann & Macler, 1986; Hirst & Wood, 1987) that a right shift of the $HbO_2$ dissociation curve leads to a reduced fraction of hypoxic cells in tumours if the oxygen supply conditions in other respects are constant. The hypoxic fraction of the KHT, RIF-1 and MLS tumours increased with increasing volume and hence decreasing pH. The relationship between $HbO_2$ saturation status and tumour pH was therefore not a direct consequence of the pH-dependence of the $HbO_2$ dissociation curve, although reduced haemoglobin affinity at acid pH may have contributed significantly.

$HbO_2$saturation status decreased and fraction of hypoxic cells increased with increasing tumour volume for the KHT, RIF-1 and MLS lines, whereas both parameters were volume-independent for the OWI line, indicating a relationship between these two parameters within tumour lines. Similarly, Vaupel *et al.* (1981) have presented data for a C3H mouse mammary adenocarcinoma line indicating a relationship between tissue $pO_2$ measured with microelectrodes and hypoxic fraction.

However, there was no correlation between $HbO_2$ saturation status and fraction of hypoxic cells across the four tumour lines. This indicates that the hypoxic fraction of a specific tumour type is not determined solely by the oxygen supply conditions; other factors may also be important, e.g. oxygen diffusivity, rate of oxygen consumption and cell survival under hypoxic stress. Oxygen diffusivity depends on tissue water content (Vaupel, 1976) and the water content may differ considerably among different tumours as revealed by NMR measurements of proton $T_1$ and $T_2$ relaxation times (Kiricuta & Simplaceanu, 1975). Rate of oxygen consumption and its dependence on oxygen and glucose availability have been found to differ considerably among tumour lines both *in vitro* and *in vivo* (Gullino, 1976; Vaupel, 1979; Sutherland, 1986). Cell survival time under hypoxic conditions depends on intrinsic properties of the tumour cells and is significantly modified by low glucose concentrations and acid pH (Steel, 1977; Wike-Hooley *et al.*, 1984; Rotin *et al.*, 1986; Freyer & Sutherland, 1986). Moreover. *in vitro* studies of multicellular spheroids also suggest that the hypoxic fraction of a tumour is not determined only by the oxygen supply conditions; spheroids of the same size from different tumour lines grown and irradiated under identical oxygen supply conditions may show significantly different hypoxic fractions (Acker *et al.*, 1984; Müller-Klieser, 1987).

Adequate, reliable methods for prediction of radioresistance caused by hypoxia and for monitoring of tumour oxygenation status during fractionated radiation therapy are highly needed in order to individualize and hence optimize clinical radiation therapy (Peter *et al.*, 1984). Cryospectrophotometric measurement of intracapillary $HbO_2$ saturations in tumour biopsies will probably have limited practical value in that respect, mainly because there is no clear relationship between $HbO_2$ saturation status and fraction of radiobiologically hypoxic cells in tumours (Figure 9). Moreover, different biopsies from the same tumour may show significantly different $HbO_2$ frequency distributions due to pronounced inhomogeneities in oxygen supply conditions within tumours in man (Müller-Klieser *et al.*, 1981; Wendling *et al.*, 1985). However, it cannot be excluded that the cryospectrophotometric method may be of some value in clinical radiation therapy if used in combination with other methods for prediction of radioresistance and monitoring of reoxygenation or used to confirm the effectiveness of physiological interventions designed to change the oxygen carrying capacity of the blood in tumours.

The cryospectrophotometric technique has, on the other hand, a significant potential in experimental studies of tumour oxygenation since it can be used to provide quantitative information on the $HbO_2$ saturation in any capillary in a tumour. This unique feature makes the technique very powerful in studies of relationships between oxygen supply and important biological phenomena such as cell proliferation and differentiation, malignant progression, and development of hypoxia and necrosis, relationships that are not well characterized and understood for tumours *in vivo.*

This study was supported by The Norwegian Cancer Society, The Fulbright Program and Grants No. CA-20329 and CA-11198 from The National Cancer Institute. We are grateful to Dr Carl Honig, Department of Physiology, University of Rochester Medical Center, for the use of his laboratory and cryospectrophotometer, and to Dr Robert Bryant, Department of Biophysics, University of Rochester Medical Center, for the use of the NMR facility and the CSI spectrometer. We also wish to thank Ms Marianne Rofstad for her excellent technical assistance and Mary LeRoy-Jacobs and Lynn Palmiere for typing the manuscript.

### References

ACKER, H., CARLSSON, J., DURAND, R. & SUTHERLAND, R.M. (1984). *Spheroids in Cancer Research: Methods and Perspectives.* Springer-Verlag: New York.

BLITZER, P.H., WANG, C.C. & SUIT, H.D. (1984). Blood pressure and haemoglobin concentration: Multivariate analysis of local control after irradiation for head and neck cancer. *Int. J. Radiat. Oncol. Biol. Phys.*, **10** (Suppl. II), 98.

BUSH, R.S., JENKIN, R.D.T., ALLT, W.E.C. & 4 others (1978). Definitive evidence for hypoxic cells influencing cure in cancer therapy. *Br. J. Cancer*, **37** (Suppl. III), 302.

CALDERWOOD, S.K. & DICKSON, J.A. (1982). Inhibition of tumour blood flow at high blood sugar levels: Effects on tumour pH and hyperthermia. *Natl Cancer Inst. Monogr.*, **61**, 221.

COURTENAY, V.D. & MILLS, J. (1978). An *in vitro* colony assay for human tumours grown in immune-suppressed mice and treated *in vivo* with cytotoxic agents. *Br. J. Cancer*, **37**, 261.

DEGNER, F. & GAYESKI, T.E.J. (1988). A comparison of a four wavelength analysis and multicomponent wavelength analysis applied to determination of haemoglobin saturation. *Adv. Exp. Med. Biol.* (in press).

DISCHE, S., ANDERSON, P.J., SEALY, R. & WATSON, E.R. (1983). Carcinoma of the cervix – Anaemia, radiotherapy and hyperbaric oxygen. *Br. J. Radiol.*, **56**, 251.

FENTON, B.M., GAYESKI, T.E.J., ROFSTAD, E.K. & SUTHERLAND, R.M. (1988). Cryospectrophotometric determination of $HbO_2$ saturation in microvessels, *Am. J. Physiol.* (in press).

FOLKMAN, J. & COTRAN, R. (1976). Relation of vascular proliferation to tumour growth. *Int. Rev. Exp. Pathol.*, **16**, 207.

FREYER, J.P. & SUTHERLAND, R.M. (1986). Regulation of growth saturation and development of necrosis in EMT6/Ro multicellular spheroids by the glucose and oxygen supply. *Cancer Res.*, **46**, 3504.

GADIAN, D.G., RADDA, G.K., DAWSON, M.J. & DOUGLAS, R.W. (1982). pH: Measurements of cardiac and skeletal muscle using $^{31}$P-NMR. In *Intracellular pH: Its Measurement, Regulation and Utilization in Cellular Functions*, Nuccitelli & Deamer (eds) p. 61. Alan R. Liss: New York.

GAYESKI, T.E.J. (1981). *A Cryogenic Microspectrophotometric Method for Measuring Myoglobin Saturation in Subcellular Volumes: Application to Resting Dog Gracilis Muscle.* Ph.D. Dissertation, University of Rochester: Rochester, NY.

GRAY, L.H. & STEADMAN, J.M. (1964). Determination of the oxyhaemoglobin dissociation curves for mouse and rat blood. *J. Physiol.*, **175**, 161.

GRUNEWALD, W.A. & LÜBBERS, D.W. (1975). Die Bestimmung der intracapillären $HbO_2$-Sätigung mit einer kryo-mikrofotometrischen Methode angewandt am Myocard des Kaninchens. *Pflügers Arch. Eur. J. Physiol.*, **353**, 255.

GRUNEWALD, W.A. & LÜBBERS, D.W. (1976). Kryomicrophotometry as a method for analyzing the intracapillary $HbO_2$ saturation of organs under different $O_2$ supply conditions, *Adv. Exp. Med. Biol.*, **75**, 55.

GULLINO, P.M. (1976). *In vivo* utilization of oxygen and glucose by neoplastic tissue. *Adv. Exp. Med. Biol.*, **75**, 521.

HIRST, D.G. (1986). Oxygen delivery to tumors. *Int. J. Radiat. Oncol. Biol. Phys.*, **12**, 1271.

HIRST, D.G. & WOOD, P.J. (1987). The influence of haemoglobin affinity for oxygen on tumour radiosensitivity. *Br. J. Cancer*, **55**, 487.

HOFFMANN, J. & LÜBBERS, D.W. (1985). Quantitative analysis of reflection spectra: Evaluation of simulated reflection spectra. *Adv. Exp. Med. Biol.*, **191**, 889.

HOFFMANN, J., WODICK, R., HANNEBAUER, F. & LÜBBERS, D.W. (1984). Quantitative analysis of reflection spectra of the surface of the guinea pig brain. *Adv. Exp. Med. Biol.*, **169**, 831.

JAIN, R.K., SHAH, S.A. & FINNEY, P. (1984). Continuous noninvasive monitoring of pH and temperature in rat Walker 256 carcinoma during normoglycemia and hyperglycemia. *J. Natl Cancer Inst.*, **73**, 429.

KIRICUTA, I.C. & SIMPLACEANU, V. (1975). Tissue water content and nuclear magnetic resonance in normal and tumor tissues. *Cancer Res.*, **36**, 1164.

KUBELKA, P. & MUNK, F. (1931). Ein Betrag zur Optik der Farbanstriche. *Z. Techn. Phys.*, **11a**, 593.

LÜBBERS, D.W. & WODICK, R. (1969). The examination of multicomponent systems in biological materials by means of a rapid scanning photometer. *Appl. Optics*, **8**, 1055.

MÜLLER-KLIESER, W. (1987). Multicellular spheroids: A review on cellular aggregates in cancer research. *J. Cancer Res. Clin. Oncol.*, **113**, 101.

MÜLLER-KLIESER, W., VAUPEL, P. & MANZ, R. (1983). Tumour oxygenation under normobaric and hyperbaric conditions. *Br. J. Radiol.*, **56**, 559.

MÜLLER-KLIESER, W., VAUPEL, P., MANZ, R. & GRUNEWALD, W.A. (1980). Intracapillary oxyhaemoglobin saturation in malignant tumours with central or peripheral blood supply. *Eur. J. Cancer*, **16**, 195.

MÜLLER-KLIESER, W., VAUPEL, P., MANZ, R. & SCHMIDSEDER, R. (1981). Intracapillary oxyhaemoglobin saturation of malignant tumours in humans. *Int. J. Radiat. Oncol. Biol. Phys.*, **7**, 1397.

NG, T.C., EVANOCHKO, W.T., HIRAMOTO, R.N. & 6 others (1982). $^{31}$P NMR spectroscopy of *in vivo* tumors. *J. Magn. Reson.*, **49**, 271.

OVERGAARD, J., SAND HANSEN, H., JÖRGENSEN, K. & HJELM HANSEN, M. (1986). Primary radiotherapy of larynx and pharynx carcinoma – An analysis of some factors influencing local control and survival. *Int. J. Radiat. Oncol. Biol. Phys.*, **12**, 515.

PETERS, L.J., HOPWOOD, L.E., RODNEY WITHERS, H. & SUIT, H.D. (1984). Predictive assays of tumour radiocurability. *Cancer Treat. Symp.*, **1**, 67.

PITTMAN, R.N. (1986). Microvessel blood oxygenation measurement techniques. In *Microcirculatory Technology*, Baker & Nastuk (eds) p. 367. Academic Press: Orlando.

RENEAU, D.D. & SILVER, I.A. (1978). Some effects of high altitude and polycythaemia on oxygen delivery. *Adv. Exp. Med. Biol.*, **94**, 245.

REVESZ, L. & BALMUKHANOV, S.B. (1987). Anaemia as a prognostic factor for the therapeutic effect of radiosensitizers. *Int. J. Radiat. Biol.*, **51**, 591.

ROFSTAD, E.K. (1981). Radiation response of the cells of a human malignant melanoma xenograft. Effect of hypoxic cell radiosensitizers. *Radiat. Res.*, **87**, 670.

ROFSTAD, E.K., DEMUTH, P. & SUTHERLAND, R.M. (1988). $^{31}P$ NMR spectroscopy measurements of human ovarian carcinoma xenografts: Relationship to tumour volume, growth rate, necrotic fraction and differentiation status. *Radiother. Oncol.* (in press).

ROFSTAD, E.K. & SUTHERLAND, R.M. (1988). Radiation sensitivity of human ovarian carcinoma cell lines *in vitro*: Effects of growth factors and hormones, basement membrane, and intercellular contact. *Int. J. Radiat. Oncol. Biol. Phys.* (in press).

ROTIN, D., ROBINSON, B. & TANNOCK, I.F. (1986). Influence of hypoxia and an acidic environment on the metabolism and viability of cultured cells: Potential implications for cell death in tumors. *Cancer Res.*, **46**, 2821.

SIEMANN, D.W. & MACLER, L.M. (1986). Tumor radiosensitization through reductions in haemoglobin affinity. *Int. J. Radiat. Oncol. Biol. Phys.*, **12**, 1295.

SOLESVIK, O.V., ROFSTAD, E.K. & BRUSTAD, T. (1982). Vascular structure of five human malignant melanomas grown in athymic nude mice. *Br. J. Cancer*, **46**, 557.

STEEL, G.G. (1977). *Growth Kinetics of Tumours*. Clarendon Press: Oxford.

STORM, F.K. (1983). *Hyperthermia in Cancer Therapy*. G.K. Hall Medical Publishers: Boston.

SUTHERLAND, R.M. (1986). Importance of critical metabolites and cellular interactions in the biology of microregions of tumors. *Cancer*, **58**, 1668.

SUTHERLAND, R.M., DEGNER, F.L., ROFSTAD, E.K. & 5 others (1987). Measurement of tumor oxygenation and energy status using cryospectrophotometry and $^{31}P$ magnetic resonance spectroscopy. In *Prediction of Tumor Treatment Response*, p. E11. Omnipress: Madison.

TANNOCK, I.F. (1970). Population kinetics of carcinoma cells, capillary endothelial cells, and fibroblasts in a transplanted mouse mammary tumour. *Cancer Res.*, **30**, 2470.

THOMLINSON, R.H. & GRAY, L.H. (1955). The histological structure of some human lung cancers and the possible implications for radiotherapy. *Br. J. Cancer*, **9**, 539.

THOMSON, J.E. & RAUTH, A.M. (1974). An *in vitro* assay to measure the viability of KHT tumor cells not previously exposed to culture conditions. *Radiat. Res.*, **58**, 262.

TWENTYMAN, P.R., BROWN, J.M., GRAY, J.W., FRANKO, A.J., SCOLES, M.A. & KALLMAN, R.F. (1980). A new mouse tumor model system (RIF-1) for comparison of end-point studies. *J. Natl Cancer Inst.*, **64**, 594.

URANO, M., GERWECK, L.E., EPSTEIN, R., CUNNINGHAM, M. & SUIT, H.D. (1980). Response of a spontaneous murine tumor to hyperthermia: Factors which modify the thermal response *in vivo*. *Radiat. Res.*, **83**, 312.

VAN ASSENDELFT, O.W. (1970). *Spectrophotometry of Haemoglobin Derivatives*. Thomas: Springfield, IL.

VAUPEL, P. (1976). Effect of percentual water content in tissues and liquids on the diffusion coefficients of $O_2$, $CO_2$, $N_2$, and $H_2$. *Pflügers Arch. Eur. J. Physiol.*, **361**, 201.

VAUPEL, P. (1977). Hypoxia in neoplastic tissue. *Microvasc. Res.*, **13**, 399.

VAUPEL, P. (1979). Oxygen supply to malignant tumors. In *Tumor Blood Circulation: Angiogenesis, Vascular Morphology and Blood Flow of Experimental and Human Tumors*, Peterson (ed) p. 143. CRC Press, Inc.: Boca Raton.

VAUPEL, P., FRINAK, S. & BICHER, H.I. (1981). Heterogeneous oxygen partial pressure and pH distribution in C3H mouse mammary adenocarcinoma. *Cancer Res.*, **41**, 2008.

VAUPEL, P., GRUNEWALD, W.A., MANZ, R. & SOWA, W. (1978). Intracapillary $HbO_2$ saturation in tumor tissue of DS-carcinosarcoma during normoxia. *Adv. Exp. Med. Biol.*, **94**, 367.

VAUPEL, P., MANZ, R., MÜLLER-KLIESER, W. & GRUNEWALD, W.A. (1979). Intracapillary $HbO_2$ saturation in malignant tumors during normoxia and hyperoxia. *Microvasc. Res.*, **17**, 181.

WENDLING, P., MANZ, R., THEWS, G. & VAUPEL, P. (1984). Heterogeneous oxygenation of rectal carcinomas in humans: A critical parameter for preoperative irradiation? *Adv. Exp. Med. Biol.*, **180**, 293.

WIKE-HOOLEY, J.L., HAVEMAN, J. & REINHOLD, H.S. (1984). The relevance of tumor pH to the treatment of malignant disease. *Radiother. Oncol.*, **2**, 343.

ZANDER, R. & SCHMID-SCHÖNBEIN, H. (1972). Influence of intracellular convection on the oxygen release by human erythrocytes. *Pflügers Arch. Eur. J. Physiol.*, **335**, 58.